# Hierarchical distance sampling to estimate population sizes of common lizards across a desert ecoregion

**DOI:** 10.1002/ece3.4780

**Published:** 2019-02-20

**Authors:** Brett J. Furnas, D. Scott Newton, Griffin D. Capehart, Cameron W. Barrows

**Affiliations:** ^1^ Wildlife Investigations Laboratory California Department of Fish and Wildlife Rancho Cordova California; ^2^ Wildlife Branch California Department of Fish and Wildlife Sacramento California; ^3^ Department of Wildlife Humboldt State University Arcata California; ^4^ Center for Conservation Biology University of California Riverside Riverside California

**Keywords:** *Aspidoscelis tigris*, *Callisaurus draconoides*, climate change, density, land use, Mojave Desert, monitoring, *Uta stansburiana*

## Abstract

Multispecies wildlife monitoring across large geographical regions is important for effective conservation planning in response to expected impacts from climate change and land use. Unlike many species of birds, mammals, and amphibians which can be efficiently sampled using automated sensors including cameras and sound recorders, reptiles are often much more challenging to detect, in part because of their typically cryptic behavior and generally small body sizes. Although many lizard species are more active during the day which makes them easier to detect using visual encounter surveys, they may be unavailable for sampling during certain periods of the day or year due to their sensitivity to temperature. In recognition of these sampling challenges, we demonstrate application of a recent innovation in distance sampling that adjusts for temporary emigration between repeat survey visits. We used transect surveys to survey lizards at 229 sites throughout the Mojave Desert in California, USA, 2016. We estimated a total population size of 82 million (90% CI: 65–99 million) for the three most common species of lizards across this 66,830 km^2^ ecoregion. We mapped how density at the 1‐km^2^ scale was predicted to vary with vegetation cover and human development. We validated these results against independent surveys from the southern portion of our study area. Our methods and results demonstrate how multispecies monitoring programs spanning arid ecoregions can better incorporate information about reptiles.

## 1 INTRODUCTION

Climate change and human land use pose major challenges for biological conservation globally (Jetz, Wilcove, & Dobson, 2007; Newbold et al., 2015; Walther et al., 2002). For this reason, agencies and organizations are increasingly investing in long‐term, large‐scale, multispecies wildlife monitoring programs (Ahumada, Hurtado, & Lizcano, 2013; Furnas & Callas, 2015; Nielsen, Haughland, Bayne, & Schieck, 2009). Unlike many species of birds, mammals, and amphibians which can be sampled using automated survey equipment including cameras and sound recorders (Blumstein et al.., 2011; Burton et al., 2015), reptiles are often much more difficult to detect, in part because of their typically cryptic behavior and generally small body sizes (Griffiths, Foster, Wilkinson, & Sewell, 2015). Therefore, there is a need for improved survey and analytical methods for estimating occupancy, diversity, and abundance of reptiles for inclusion in multispecies monitoring projects (Gibbons et al., 2000; Griffiths et al., 2015).

Diurnal lizards may be particularly vulnerable to climate change in desert ecoregions throughout the world, in part because sheltering from lethal temperatures may limit foraging and reproductive activities which in turn may impact population levels (Sinervo et al., 2010; Sow, Martínez‐Freiría, Dieng, Fahd, & Brito, 2014; Vale & Brito, 2015). Ecological niche models and evidence of recent extinctions suggest that lizards are already exceeding physiological limits in North American deserts (Barrows, 2011; Sinervo et al., 2010). Furthermore, lizards may be good indicators of ecological change in arid environments with respect to both climate and land use (Barrows, Hoines, Vamstad, Murphy‐Mariscal, & K, Lalumiere, and J. Heintz., 2016). Specifically, researchers have found lizard diversity or abundance to be associated, either positively or negatively, with rainfall, temperature changes along elevation gradients, fluctuations in arthropod abundance, spatial heterogeneity of vegetation communities, livestock grazing, land degradation due to mining, urban development, and numerous other factors (Ackley, Wu, Angilletra, Myint, & Sullivan, 2015; McCain, 2010; Thompson, Thompson, & Withers, 2008; Waudby & Petit, 2015; Whitford & Creusere, 1977).

Compared to other desert reptiles, many lizard species tend to be more active during the day which makes them easier to detect using visual encounter surveys (Parker & Pianka, 1975; Pianka, 1970; Pianka & Parker, 1975). However, accurate population or distribution assessments of any wildlife species typically require robust analytical methods that explicitly model site‐ and survey‐level heterogeneity in detection probability (Kery & Royle, 2016; MacKenzie et al., 2006). A related problem is that lizards may be unavailable for sampling during parts of the day or year that are either too cold or too hot (Jacome‐Flores, Blazquez, Sosa, & Maya, 2015; Whitford & Creusere, 1977; Wone & Beauchamp, 2003). This issue necessitates more complex hierarchical models that allow for the assumption that some proportion of a population is not available for sampling during surveys (Chandler, Royle, & D. I. King., 2011). The failure to consider imperfect availability may lead to underestimation of density as has been noted for distance sampling of lizards (Rodda & Campbell, 2002; Smolensky & Fitzgerald, 2010). The issue of availability bias is not unique to lizards; it is also a problem for distance sampling of sharks (Nykanen et al., 2018) marine mammals (Danilewicz et al., 2010), terrestrial mammals (Poole, Cuyler, & Nymand, 2013), birds (Gale et al.., 2009), and other reptiles (Couturier, Cheylan, Bertolero, Astruc, & Besnard, 2013).

Perhaps due to the difficulties in surveying reptiles, there are very few estimates of lizard density from the Mojave Desert in North America, and those studies that have been attempted were for small areas within this region (Kaufmann, 1982; Turner, Medica, Lannom, & Hoddenbach, 1969). To address this information gap, we provide the first robust estimates of density and total population size for three species of common lizards [Common Side‐blotched Lizard (*Uta stansburiana*), Western Whiptail (*Aspidoscelis tigris*), and Zebra‐tailed Lizard (*Callisaurus draconoides*)] throughout the Mojave Desert within the State of California, USA (66,830 km^2^). We did this by adding visual encounter surveys along transects to a program of multispecies surveys (i.e., birds and mammals) at widespread sampling sites throughout the study area. We demonstrate application of a recent innovation in distance sampling that adjusts for temporary emigration between repeat survey visits (Chandler et al., 2011; Kery & Royle, 2016). We show how this survey and analytical approach combined with model‐based inference (Gregoire, 1998) across the study area facilitates efficient estimation of population size as well as coarse‐scale mapping of density for each species. We validate our results through comparison with independent surveys conducted in the southern portion of the study area in the same year. We discuss how our results could be used to inform region‐level planning in response to expected climate change and land use impacts, and consider the value of common lizards as an indicator of those stressors in an arid environment.

## 2 STUDY AREA

Our 66,830 km^2^ study area includes the Mojave Desert ecoregion within California. Average elevation is 796 m which is lower than the Great Basin to the north and higher than the Sonoran Desert to the south; however, elevations in the study area range from −83 m in Death Valley to 2,405 m along borders with adjacent mountain ranges. Average annual precipitation over the past 30 years ranged from 46 to 695 mm ($\overset{¯}{x}$ = 146 mm). Vegetation communities included those dominated by *Larrea tridentata* at lower elevations, *Yucca brevifolia *at middle elevations, and *Juniperus osteosperma* at higher elevations. Based on multispecies surveys, we conducted at the same sites, the most common wildlife were black‐tailed jackrabbit (*Lepus californicus*), kit fox (*Vulpes macrotis*), and coyote (*Canis latrans*) among mammals >0.5 kg, and black‐throated sparrow (*Amphispiza bilineata*), common raven (*Corvus corax*) and horned lark (*Eremophila alpestris*) among passerine birds (California Department of Fish and Wildlife, unpublished data). Military bases covered 16% of the study area, whereas nonmilitary federal government lands covered an additional 63%, including 19,700 km^2^ of designated wilderness that receive a higher level of protection than other federal lands. Most of the wilderness lands were in the eastern half of the study area. Besides military exercises, increasing development mostly in the western part of the study area, agriculture, surface mining, off‐road vehicle recreation, and renewable energy development are potential activities impacting lizards and other wildlife.

## 3 METHODS

### 3.1 Study design

**Figure 1:**
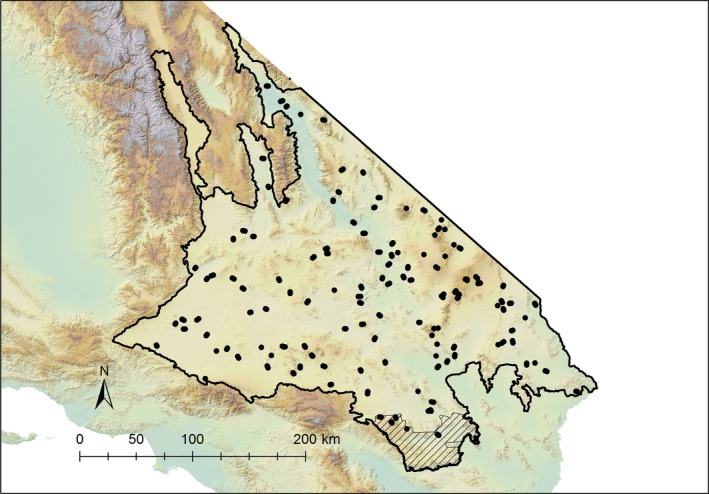
Locations of visual encounter transect surveys which occurred in the Mojave Desert within California, USA, April–July 2016. Shaded area on southern boundary represents the portion of Joshua Tree National Park that lies within the study area. We used independent surveys from this park to validate our model predictions of lizard density based on the transect surveys

We surveyed 229 sites throughout the study area between April and July 2016 (Figure 1). We determined survey locations by first selecting a spatially balanced random sample of hexagons from the USDA Forest Inventory and Analysis program's hexagon grid (Bechtold & Patterson, 2005, hexagon radius is ~2.6 km). We then randomly selected 1–3 survey locations within each hexagon, which were spaced by 1–2 km apart and stratified by vegetative community based on the National Vegetation Classification System (Sawyer, Keeler‐Wolf, & Evans, 2009, Supporting Information Table S1). In some cases, we were not able to gain access to a particular hexagon or site within a hexagon. In those instances, we adopted an iterative process, whereby we either selected a nearby hexagon or selected the next site from a sequential list of random locations from a hexagon. We did not explicitly address these site selection details in our density estimation methods because our modeling did not assume random site selection (Gregoire, 1998). However, model‐based inference requires sampling over the range of conditions used in extrapolation. We excluded sampling from the Owens Valley, shown as the northwest appendage in our study area, due to logistical limitations (Figure 1), but we did not expect this omission to substantially bias our population estimates because this subregion constituted <3% of the study area. We also excluded sampling at elevations above 1,630 m, but these elevations amounted to only 0.75% of the study area.

### 3.2 Wildlife surveys

A two‐person team conducted a visual encounter survey for lizards and other taxa along an approximately 400‐m transect at each site. Two overlapping approximately 200‐m transect legs were established at each site. They generally formed a cross‐centered on the vegetation type from our stratified selection of survey sites. However, alternative configurations (e.g., T‐junction, single long transect) were sometimes adopted instead, when the terrain prohibited the desired transect configuration. One person devoted their full attention to surveying all lizard species and estimating initial perpendicular distance from the transect to each observation with the aid of a laser range finder. Binoculars were not used for surveying lizards. A second person who was also surveying for birds and other taxa recorded all the lizard detections, observed by the first person, on a data sheet. Surveys were repeated on up to three occasions (3 visits = 212 sites, 2 visits = 16 sites, 1 visit = 1 site) one to four weeks apart during visits when surveys for other taxa occurred (e.g., sounds recorders for birds and bats, camera stations for mammals >0.5 kg). However, the visual encounter surveys were always conducted first to avoid flushing of lizards during the other survey activities. No survey information was collected when travelling between the end point of the first transect leg and the start point of the second transect leg. An ambient air temperature measurement was made before commencing each transect leg. This measurement was made 30–35 cm above the soil surface using a thermometer that was shaded from the sun using either the surveyor's body or a clipboard.

We used the repeated surveys to create detection histories for the three most common lizard species (*A. tigris*, *U. stansburiana*, and *C. draconoides*). Specifically, we tallied the total number of adult lizards observed along the transect at each site on each survey occasion within each 1‐m distance increment out to 13 m. We chose this truncation threshold consistent with recommendations for distance sampling because it was the distance within which 95% of our observations were made (Buckland et al., 2001). Furthermore, to address unintentional “heaping” of distance observations in the 5‐m and 10‐m categories, we reclassified distances in to 6 heterogeneous categories (Buckland et al., 2001): 0–0.5 m, 0.5–1.5 m, 1.5–2.5 m, 2.5–3.5 m, 3.5–6.5 m, and 6.5–13.5 m. We acknowledge a small area of survey overlap because of the crossing of transect legs. Given the general infrequency of lizard detections (Supporting Information Table S2), we believe any potential bias due to double counting was low. However, we removed the area of overlap ([2 × 13.5 m]^2^) from the area denominator used in our density calculations.

### 3.3 Site and survey covariates

We expected lizard densities to vary with environmental conditions across the study area. Vegetation communities in the Mojave Desert are strongly associated with elevation gradients of temperature and precipitation (Schoenherr, 1992). Higher elevations tend to support greater vegetation cover and floristic diversity (Sawyer et al., 2009). Some researchers have found an association between lizard diversity and intermediate vegetation cover often found at middle elevations in arid environments (McCain, 2010). Others have found vegetation composition or density to be an important predictor of lizard occurrence in deserts (Heaton, Kiester, & Meyers, 2006). Therefore, we used a 1‐km raster of a normalized difference vegetation index (NDVI) from June 2016 to represent vegetation cover to predict lizard abundances at survey sites (Cohen & Goward, 2004). Other researchers have shown this index to be a good proxy for vegetation cover for modeling the distribution of biodiversity in arid environments (Dubinin, Svoray, Dorman, & Perevolotsky, 2018; Macías‐Duarte, Panjabi, Pool, Ruvalcaba‐Ortega, & Levandoski, 2018). We also decided to use NDVI because we had access to its values across the entire study area which facilitated extrapolation of population size using model‐based inference. To augment the value of NDVI as an indicator of vegetation cover, we coerced all negative values generally indicative of water or bare earth to zero (Almanza, Jerrett, Dunton, Seto, & Pentz, 2012). We did not include elevation in the modeling because it was highly correlated with NDVI (*r* = 0.69).

Lizards have been shown to be negatively impacted by and to be good indicators of anthropogenic development (Ackley et al., 2015; Thompson et al., 2008; Waudby & Petit, 2015). To predict lizard abundances at survey sites, we used a 30‐m raster land use land cover layer, which we aggregated as a binary raster (1 or 0) to represent either urban, agricultural, transportation, or mining‐related development or none of these categories from the most recent year available (CalFIRE, 2015). Finally, lizard activity is strongly affected by temperature (Huey & Pianka, 2018; McCain, 2010). We used the average of the two ambient temperature measurements made during each survey to represent average temperature as a survey‐level covariate predicting lizard activity.

We rescaled the two site‐level, abundance covariates (NDVI and development) to their average values within a 1‐km raster grid (66,812 cells) covering the entire study area. Therefore, NDVI was interpreted as average vegetation cover within 1 km^2^ and development as the total proportion of 1 km^2^ in one of the development categories considered. We chose the 1‐km scale because it matched the minimum distance between survey sites in the same sampling hexagon, and represented landscape conditions appropriate for extrapolating coarse‐scale average abundances across a large region. The grid also allowed us to use model‐based inference (Gregoire, 1998) to predict lizard densities throughout the study area instead of relying on the representativeness of our survey locations as a stratified random sample. We calculated the values of site‐level covariates at survey sites by intersecting the centroid of the transect at each site with the 1‐km grids of NDVI and development. Lastly, we standardized all covariates.

### 3.4 Hierarchical distance sampling

Distance sampling for transects is a method for modeling how detection probability [*g*(*x*)] of wildlife declines with perpendicular distance (*x*) from an observer in order to get an unbiased estimate of density. This is achieved by upwardly adjusting the raw survey counts by dividing them by a measure of total detection probability calculated by integrating *g*(*x*) across the width of a transect (Buckland et al., 2001). A core assumption of classical distance sampling is perfect detection along the center line of transect surveys. A recent innovation in hierarchical modeling allows relaxation of this assumption via temporary emigration between repeat survey occasions (Kery & Royle, 2016). Individuals sampled from an open population under this modeling approach can be viewed as unavailable for sampling during some parts of a sampling season (Chandler et al., 2011). This statistical assumption matches the ecology of lizards which through thermoregulation behaviors were expected to be below ground in burrows or under other cover during periods of the season, days, or portions of days that were either too cold or too hot (Jacome‐Flores et al., 2015; Whitford & Creusere, 1977; Wone & Beauchamp, 2003). A hierarchical modeling structure allows generalization of the abundance, availability, and detection probability [e.g., *g*(*x*)] processes such that each can be modeled to vary with covariates by site and survey occasion and with respect to assumptions about probability distributions (Sillett, Chandler, Royle, Kery, & Morrison, 2012).

The hierarchical distance sampling model we used for single species included three levels for sites *i* and survey occasions *k*:

*Level 1*: Total abundance *M_i_* (latent state) and density *D_i_* (derived quantity).
*D_i_* = *M_i_*/*A_i_*, where *A* are the effective survey areas of transects at sites
*M_i_* ~ Negative Binomial (*λ_i_*,*γ*), where *λ* are expected abundances and *γ* is a measure of dispersion
  log(*λ_i_*) = linear model of site covariates
*Level 2*: Available abundance *N_i,k_* (latent state).
  logit(*N_i,k_*)~Binomial(*M_i_*, *ϕ_i,k_*)
*ϕ_i,k_* = linear model of survey covariates
*Level 3*: Detection function.
*y_i,k_* is a vector of observations by distance class
*y_i,k_* ~ Multinomial(*N_i,k_*, *π_i,k_*)
*π_i,k_* = *f*(*σ*), where *f* is a function of a distance decay parameter *σ*.

Greater detail about the model is described elsewhere (Kery & Royle, 2016, chapter 9). We assumed a negative exponential function for *f*(*σ*) = e^−^ *^σ^* ^*^ *^d^*, where *d* was the distance measurement for an observation. We did not include a covariate model allowing *σ* to vary with site or survey occasion. However, we compared our final models to these same models including NDVI as a covariate on *σ* to rule out the possibility that any associations we found between NDVI and abundance may have been confounded by detection probability varying with vegetation cover.

We fit distance sampling models via maximum likelihood estimation using the *gdistsamp* function in the *unmarked* package (Fiske & Chandler, 2011) for the R programming language (R Development Core Team, 2017). We evaluated NDVI and anthropogenic development covariates in the Level 1 abundance model component. We also evaluated a quadratic term of NDVI to evaluate a possible unimodal relationship. Based on our expectation of lizards being under cover during periods of the day or season that were either too cold or too hot, we always included ambient temperature and sometimes also its quadratic term in the Level 2 availability model component.

We used Akaike's information criterion (AIC) and multimodel inference principles to evaluate models including all six combinations of the abundance covariates and an intercept term which was always included (Table 1, Burnham & Anderson, 2002). Prior to fitting abundance models, we used model selection for an intercept‐only abundance model to evaluate whether both temperature and its quadratic term were appropriate covariates in the availability model component for each species.

**Table 1:** Candidate models for hierarchical distance sampling for common lizards for the Mojave Desert within California, USA, April–July 2016

Covariates^a^
*Abundance model component*
dev + ndvi + ndvi^2^
dev + ndvi
ndvi + ndvi^2^
dev
ndvi
Null
*Availability model component*
temp + temp^2^
temp
Null
*Detection model component*
Null

a dev represented the proportion of the 1‐km^2^ surrounding a survey site that was developed (i.e., urban, agriculture, mining). ndvi represents the average normalized difference vegetation index for the 1‐km^2^ area. temp represented air temperature measured on‐site during surveys.

We converted abundance estimates to density by dividing abundance from each site in the model by the effective sampling area of each transect (i.e., 2 × 13.5 m × transect length – area of transect overlap). This varied because transects varied in actual length ($\overset{¯}{x}$ = 390 m, *SD* = 40 m). We applied model‐based inference to extrapolate density across the entire study area by calculating predicted density of each species at each 1‐km grid location based on the covariate values at those locations (Furnas & McGrann, 2018; Gregoire, 1998). We used model weights of the top models (i.e., AIC weights sum to 0.95, Burnham & Anderson, 2002) to get model averaged density predictions at each grid location (Cade, 2015). This allowed us to map spatial variation in density for each species throughout the study area, and to calculate average density for the study area for each species by taking the average of the point estimates from all the 1‐km grid locations. We then computed population sizes by multiplying these averages by the total area of the study area.

We used parametric bootstrapping (20,000 iterations) to get confidence intervals for our density and population size estimates in a fashion that accounted for the multiple sources of uncertainty in our modeling (Efron, 1982). Specifically, for each model, we resampled all abundance model component parameters from their estimates assuming a multivariate normal distribution of the variance‐covariance matrix from maximum likelihood estimation. A problem arising in this step was that sampled parameter values for the quadratic term on NDVI were sometimes positive, or insufficiently negative, leading to nonsensically large (>10^20^ lizards/km^2^) density estimates inconsistent with a unimodal relationship with NDVI (e.g., highest densities at intermediate level of vegetation cover). We would have preferred to solve this problem by using a data bootstrap to refit models at each iteration, but this approach was not feasible due to the additional computing time required. Therefore, we truncated the bootstrap resampled distribution of each model parameter by setting extreme values (either >95th percentile or <5th percentile) to those maximum and minimum thresholds.

To address the effect of survey effort on the precision of our estimates, we took a sample with replacement from the 1‐km grid equal to the sample size from our study (*n* = 229) for each bootstrap re‐sampling iteration. We calculated density at those locations using the resampled model parameter values from the same iteration and the covariate values at the selected locations. We then averaged those densities to get average density for the study area. We repeated bootstrapping for each model and used model weights to get a single estimate of average density for the study area for each sampling iteration (Cade, 2015). Lastly, we calculated the standard deviation of the bootstrap‐distributed densities which we treated as the appropriate standard error with which to calculate a 90% confidence interval assuming a normal distribution about the point estimate. We adopted a Type‐I error rate of 0.1 here and elsewhere in this study in congruence with a long‐term monitoring objective (Furnas & McGrann, 2018). We repeated these procedures for each species. Additional technical details on our modeling methods including the data and R code we used are provided in Supporting Information Data S3 and S4.

### 3.5 Evaluation of model fit

For all top models used to extrapolate population size, we assessed model fit by means of a parametric bootstrap goodness‐of‐fit test using the Chi‐squared test statistic (Kery & Royle, 2016, Section 7.5.4). For each test, the null hypothesis was that Chi‐squared statistic for observed survey detection data under the fitted model was equal to Chi‐squared for data generated by the model (e.g., fitted values of expected number of detections in each distance bin during each survey at each site). We estimated the probability (*p*‐val) of the observed statistic under the null hypothesis by comparing it to the distribution of the statistic for 1,000 parametric bootstrap data sets using the *parboot* function in R. We assumed good model fit if we could not reject this null hypothesis (i.e., *p*‐value ≥0.1).

### 3.6 Model validation

We compared predictions from our modeling with independent measurements of lizard density available from Joshua Tree National Park (JTNP) located in the southern portion of the study area (Figure 1) To our knowledge, these surveys were the only data available for validating our modeling. During the spring of 2016, a team of biologists and citizen scientists systematically searched eight 9‐ha sites within the portion of JTNP overlapping our Mojave Desert study area. Each site was surveyed for approximately three hours on a single occasion during which a count of adult lizards was tallied for each species. The methods which are described in greater detail in Barrows et al. (2016) were intended as an exhaustive survey due to the length of survey time and numerous surveyors present at each site, but modeling was not used to adjust for detection and availability probabilities potentially <1. At each of the eight survey sites, we compared the JTNP survey results with predicted densities at these locations from distance sampling.

## 4 RESULTS

**Figure 2:**
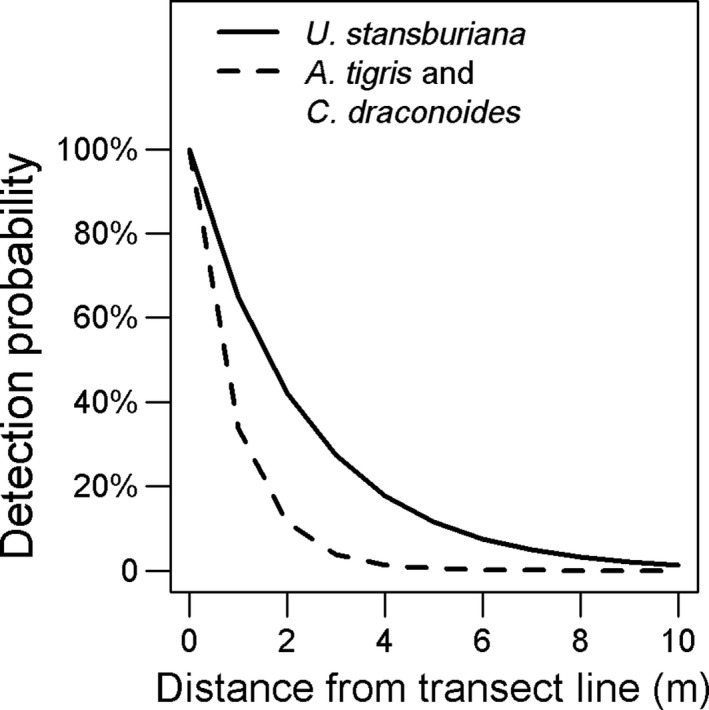
Decay in detection probability with distance during visual encounter transect surveys from the Mojave Desert within California, USA, April–July 2016. Detection probability is the average chance on seeing an individual lizard during a survey visit along a 400‐m transect if it was present along the transect during the visit

**Figure 3:**
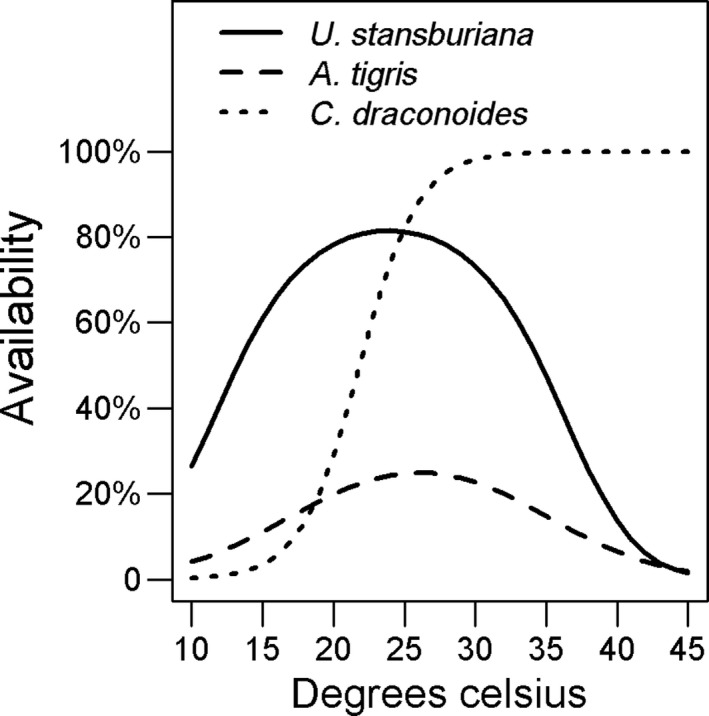
Availability of individual lizards for sampling as a function of ambient air temperature (30–35 cm above surface) along 400‐m transects surveyed from Mojave Desert within California, USA, April–July 2016. Lack of availability was interpreted as lizards seeking thermal shelter below ground during periods of cold or hot weather

We observed 12 species of lizards, but *A. tigris*, *U. stansburiana*, and *C. draconoides* accounted for 89% of all observations (Supporting Information Table S2). From distance sampling, we confirmed good model fit for all top abundance models used in model averaging (*p*‐values >> 0.1, Table 2). We also confirmed that a NDVI covariate in the detection probability model component did not improve model performance; the top abundance model with this additional covariate on detection probability always had a model weight <0.5 when compared to the same model without that covariate (Supporting Information Data S4). Detection probability declined quickly with distance from the center line of transects (Figure 2). This decline was sharper for *A. tigris* and *C. draconoides* (~20% at 2 m) than for *U. stansburiana* (~20% at 4 m). We justified inclusion of a quadratic term on temperature in the availability model components for *A. tigris* and *U. stansburiana*, but not for *C. draconoides*, because model weights for these covariate selections in a null abundance model were always >0.5 (Supporting Information Data S4). Availability for sampling of all three common species peaked at ~25°C. It declined at higher temperature for *A. tigris* and *U. stansburiana*, but remained constant above this threshold for *C. draconoides *(Figure 3). Confidence intervals on estimates of the dispersion parameter in our negative binomial formulation of the abundance model component always overlapped zero, except for *C. draconoides*. This suggests that a Poisson distribution may be a reasonable alternative assumption for *A. tigris* and *U. stansburiana*.

**Table 2:** Model selection results from hierarchical distance sampling for common lizards for the Mojave Desert within California, USA, April–July 2016

Abundance models	Model selection^a^			Parameter estimates^b^					Model fit^c^
	AIC	Delta AIC	AIC weight	Int.	dev		ndvi	ndvi^2^	*p*‐Value
*A. tigris*									
dev + ndvi	1507.57	0.00	0.27	6.510	−0.286		0.182		0.488
dev + ndvi + ndvi^2^	1507.88	0.31	0.23	6.592	−0.286		0.287	−0.099	0.491
ndvi	1508.66	1.10	0.15	6.539			0.183		0.463
ndvi + ndvi^2^	1508.88	1.31	0.14	6.624			0.293	−0.103	0.495
dev	1508.98	1.42	0.13	6.530	−0.296				0.512
Null	1510.08	2.52	0.08	6.560					0.507
*U. stansburiana*									
ndvi + ndvi^2^	1,269.45	0.00	0.70	5.935			0.949	−0.260	0.455
dev + ndvi + ndvi^2^	1,271.39	1.94	0.26	5.930	0.030		0.948	−0.261	0.468
ndvi	1,275.93	6.47							
dev + ndvi	1,277.88	8.42							
Null	1,299.85	30.39							
dev	1,301.46	32.00							
*C. draconoides*									
dev + ndvi + ndvi^2^	889.34	0.00	0.52	4.547	−0.477		−1.085	−0.737	0.508
ndvi + ndvi^2^	889.64	0.30	0.45	4.597			−1.069	−0.718	0.472
ndvi	896.18	6.84							
dev + ndvi	896.23	6.88							
Null	907.49	18.15							
dev	907.49	18.15							

a Model selection based on top models with cumulative weights summing to 0.95.

b dev represented the proportion of the 1‐km^2^ surrounding a survey site that was developed (i.e., urban, agriculture, mining). ndvi represents the average normalized difference vegetation index for the 1‐km^2^ area.

c Model fit was assessed via bootstrapping and a Chi‐square statistic testing the null hypothesis that the distribution of residuals for model fits from bootstrapping was different from that expected from our modeling. We considered a *p*‐value > 0.1 to represent good model fit.

Our proxy for vegetation cover (NDVI) and its quadratic terms were in all of the top models for *U. stansburiana *and *C. draconoides*. Our modeling predicted that density of *U. stansburiana* was greatest at a NDVI value of ~2,020 corresponding to densely vegetated areas above 1,000 m in elevation dominated by *Yucca spp*., *Juniperus osteosperma*, or *Pinus monophylla* (Supporting Information Table S1, Sawyer et al., 2009). Density of *C. draconoides* was greatest at a NDVI value of ~970 corresponding to sparsely vegetated areas below 500 m dominated by *Atriplex polycarpa*. We found a weak negative association between anthropogenic development and density of *A. tigris* versus a weak positive association for *U. stansburiana*. In contrast, we found a strong negative association for *C. draconoides*; average density throughout the study area decreased from 111 lizards in 1 km^2^ areas with <1% development to 61 lizards in 1 km^2^ areas with 5%–10% development.

**Figure 4:**
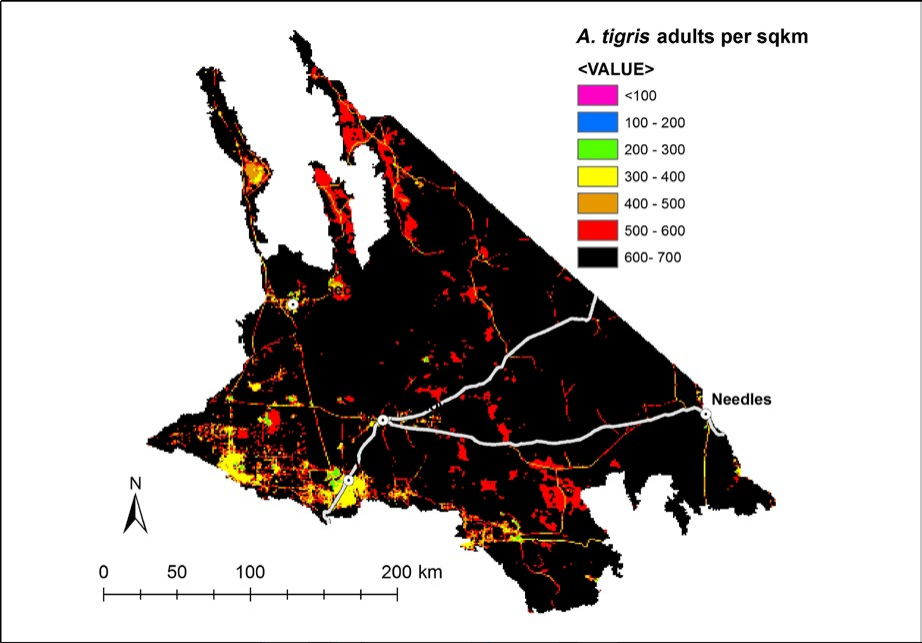
Estimated densities of Western Whiptail (*Aspidoscelis tigris*) throughout the Mojave Desert within California, USA, April–July 2016

**Figure 5:**
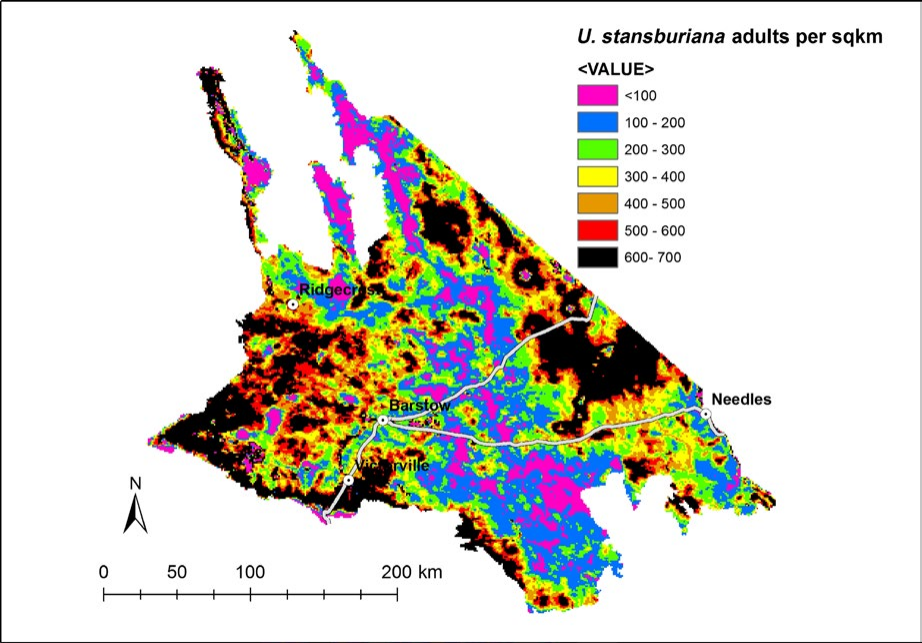
Estimated densities of Common Side‐blotched Lizard (*Uta stansburiana*) throughout the Mojave Desert within California, USA, April–July 2016

**Figure 6:**
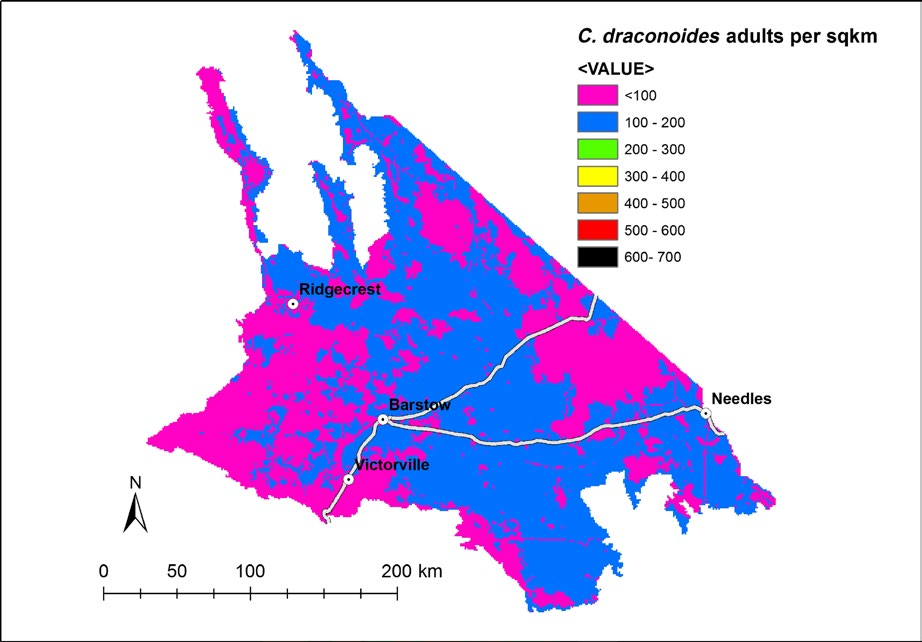
Estimated densities of Zebra‐tailed Lizard (*Callisaurus draconoides*) throughout the Mojave Desert within California, USA, April–July 2016

We obtained reasonably precise estimates of average density (and total population size) of adult lizards for each of the three most common species (Coefficients of variation [CV] = 0.13–0.27, Table 3). These results indicate that the total adult population of common lizards within the study area in mid‐2016 was 82 million (90%CI: 65–99 million), corresponding to an average density of 1,224 individuals per km^2^ (90% CI: 967–1,481 per km^2^). Our mapping of the three species indicates there was greater variation in densities within the western part of study area due to greater levels of development in this sub‐region (Figures 4, 5, 6).

**Table 3:** Estimates of density and population size of common lizards for the Mojave Desert within California, USA, April–July 2016

Species	Density (adults/km^2^)		Population size (adults)	
	Est.	90%CI	Est.	90%CI
*A. tigris*	734	547–920	49,053,220	36,556,010–61,483,600
*U. stansburiana*	388	215–561	25,930,040	14,368,450–37,491,630
*C. draconoides*	102	80–125	6,816,660	5,346,400–8,353,750
Total	1,224	967–1,481	81,799,920	64,624,610–98,975,230

Using the JTNP survey results, we validated our modeling results for *U. stansburiana* and *C. draconoides *(Table 4). Average estimated densities for these species obtained from independent methods at the eight comparison locations differed by only 16%–17%. In contrast, the average density of *A. tigris *from the JTNP surveys was 76% lower than that predicted by our modeling. For all three species, the variability of estimated density at sites was greater for the JTNP surveys than from modeling.

**Table 4:** Comparison of lizard density model estimates from the Mojave Desert, USA, 2016, with independent lizard density survey results from Joshua Tree National Park, 2016

Species	Lizards/km^2^			
	Model prediction^a^		Joshua Tree National Park surveys^b^	
	$\overset{¯}{x}$ ± *SD*	CV^c^	$\overset{¯}{x}$ ± *SD*	CV
*A. tigris*	746 ± 125	0.17	178 ± 129	0.72
*U. stansburiana*	392 ± 260	0.66	329 ± 445	1.35
*C*. *draconoides*	105 ± 57	0.55	87 ± 100	1.15

Note

Lizards were surveyed at eight Mojave Desert sites in the park. The park survey results were compared against the model predictions at these locations.

a We used 400‐m transect surveys at 229 sites throughout the Mojave Desert ecoregion within California to fit a distance sampling model predicting density at the 1‐km^2^ scale throughout the study area. The model included vegetation cover (i.e., NDVI) and anthropogenic development covariates for how density varied spatially across the study area.

b A team of wildlife biologists and citizen scientists exhaustively searched each 9‐ha site an average of 3 hr on a single occasion to get total adult counts by species, but modeling was not used to address detection probabilities potentially <1.

c Coefficient of variation: CV = *SD*/$\overset{¯}{x}$.

## 5 DISCUSSION

We provide the first robust estimates of density and population size of common lizards across a large desert ecoregion. Although the results are specific to a few species within a particular ecosystem in North America, our survey and analytical methods may be applicable to monitoring of lizard communities in arid environments throughout the world. Others have shown that the assumptions of closure and perfect detection along the center line of transects can negatively bias results from classical distance sampling (Rodda & Campbell, 2002; Ruiz de Infante Anton, Rotger, Igual, & Tavecchia, 2013; Smolensky & Fitzgerald, 2010). We demonstrated how the application of hierarchical distance sampling allows relaxation of these assumptions (Chandler et al., 2011; Kery & Royle, 2016). In particular, we found reduced availability of lizards for sampling at temperatures below and above 25°C such that we were able to correct for a substantial bias that otherwise would have left us with lower density estimates, especially for *A. tigris*.

Using the JNTP survey results, we were able to validate the accuracy of our predicted densities for *U. stansburiana *and *C. draconoides *(see Figure 3). Even though we did not fully validate our method against a known true density, the general concordance (e.g., 16%–17% lower for JTNP estimates) of both estimates for these species bolsters the credibility of our modeling and extrapolation methods. On the other hand, average density for *A. tigris* from the JTNP surveys was 76% lower than from our modeling. Consistent with the methods for the JTNP surveys, the most likely explanation for the discrepancy is the low availability of this species for sampling during a single visit. Indeed, both small and large disparities from our validation correspond closely to our estimates of availability during intermediate temperature conditions that were reflective of the JNTP surveys (25°C; 20% for *A. tigris* vs. 80% for *U. stansburiana*). Those surveys occurred during spring (March–June) and the scheduling of site visits was timed to minimize variation in temperature and maximize availability during a single visit (Barrows et al., 2016)

The ability to obtain accurate and reasonably precise population estimates of lizards across large regions will be critical for monitoring trends and identifying the effects of stressors including climate change and land use (Gibbons et al., 2000; Griffiths et al., 2015). Impacts identified to lizards may provide a good indicator of impacts to the larger ecosystem, because of this taxon's sensitivity to temperature and land use (Thompson et al., 2008; Waudby & Petit, 2015; Whitford & Creusere, 1977). Common species such as those we surveyed may disproportionately reflect ecological processes within a region, in part because they represent a large proportion of total individuals and biomass (Gaston & Fuller, 2008; Inger et al., 2015). Furthermore, monitoring common species may allow planners to develop effective conservation strategies before a species becomes endangered.

Nevertheless, we are cognizant that monitoring of common species is not a silver bullet. It can complement, but not replace, the need for specialized surveys targeting rare or localized species that may already be endangered (e.g., *Phyrnosoma mcallii*, Grant & Doherty, 2007). A need also remains to better monitor other reptile taxa that were not well surveyed by our method (e.g., snakes and *Gopherus agassizii*). This may be best achieved by a combination of survey methods each targeting different groups of species (Garden, McAlpine, Possingham, & Jones, 2007). Potential alternative survey methods include time‐constrained visual searches of quadrats (Barrows et al., 2016) pit‐fall traps (Mengak & Guynn, 1987), and environmental DNA (Kucherenko, Herman, Everham, & Urakawa, 2018).

Multispecies abundance or occupancy modeling is one potential approach to using surveys such as ours to simultaneously draw inference about both common and rare species (Iknayan, Tingley, Furnas, & Beissinger, 2014). We initially attempted to fit a Bayesian multispecies abundance model to all 12 species of lizards detected (Sollmann, Gardner, Williams, Gilbert, & Veit, 2016), but model fit was poor. We suspect this was because random effects assuming a normal distribution of separate parameter values among species failed to adequately describe differences among common and rare species. For this reason, we restricted modeling to common species using single‐species models. However, we recommend additional investigation in to the feasibility of multispecies models including alternative probability distributions governing hyperparameters (Iknayan et al., 2014) and integration of data from multiple survey methods (Garden et al., 2007; Pacifici et al., 2017).

Our modeling was intended to represent average densities at the 1‐km^2^ scale across an ecoregion. We believe this approach was appropriate for estimating population size and identifying general spatial patterns in density. However, we acknowledge that our coarse‐scale approach likely missed fine‐scale habitat selection features of importance to lizard conservation. By using spatial covariates at the 1‐km^2^ scale, we may have missed important spatial heterogeneity (i.e., rocky terrain) and climate (i.e., canyons and north facing slopes) features important to lizards that are better described at the 1–10 ha scale. Indeed, whereas comparison of our modeling with independent surveys from JTNP confirmed similar average densities for *U. stansburiana* and *C. draconoides*, there was much less variation among sites from our modeling than from the JTNP survey results (Table 4). Failure to consider appropriate scales when projecting climate change impacts can lead to potentially inaccurate conservation implications as illustrated with *Y. brevifolia* in the southern Mojave Desert (Barrows & Murphy‐Mariscal, 2012). Therefore, we recommend future integration of surveys and modeling that represents habitat conditions and population distribution at multiple spatial scales (Nichols et al., 2008; Pacifici et al., 2017). Through a double‐sampling approach, a more intensive spatial capture recapture design could be implemented at a subset of the monitoring sites where distance sampling transects occur (Dennis, Ponciano, & Taper, 2010; Royle, Chandler, Sollmann, & Gardner, 2014). Our multispecies surveys throughout an ecoregion and comparison of modeling results with the JTNP data represent a significant first step toward this goal.

By including on‐site temperature measurements in our modeling, we found that lizard activity peaked at an air temperature of about 25°C, above which it declined for *A. tigris* and *U. stansburiana *dropping to close to zero at temperatures above 40°C. These findings confirm a behavioral response of lizards to temperature. Such a pattern could mean that high “availability” in our modeling represents a heightened detection probability when lizards were more visible while basking or otherwise present in exposed locations. It would be important for a monitoring program to look for shifts in availability with respect to temperature. This is important because lizards have physiological limits above which high body temperature is lethal (Cowles & Bogert, 1944), and there is evidence that these thresholds are being increasingly exceeded in some places in North American deserts due to climate change (Sinervo et al., 2010). It is also important because higher temperatures have been shown to cause mortality in lizard embryos sheltered in nests, which suggests that impacts at this stage of the life cycle may have a greater effect on population levels than thermal impacts to adults (Levy et al., 2015).

We used a measurement of air temperature in our modeling as a proxy for the operative temperatures affecting lizard physiology and behavior (Dzialowski, 2005). We expect that direct consideration of operative temperatures would improve performance of abundance modeling (Angeli, Lundgren, Pollock, Hillis‐Starr, & Fitzgerald, 2018). Additionally, incorporation of daily maximum temperature models available at the 4‐km scale across the entire study area (Daly et al., 2008) in to hierarchical modeling of wildlife survey data may facilitate greater inference about behavioral responses of wildlife to climate change (McGrann & Furnas, 2016).

Interestingly, *A. tigris* was the most abundant lizard, but it was also more difficult to consistently detect than the other two common species. The detectability of this species dropped steeply with distance (Figure 2) and it had the lower overall availability for sampling (Figure 3). We posit that the lower detectability and availability of this species may be largely attributed to its spatial and behavioral autecology. *A. tigris* is an active forager with a large home range and spends less time basking, and more time actively shuttling between microhabitats than the two more territorial species (*U. stansburiana, C. draconoides*) (Parker & Pianka, 1975; Turner et al., 1969).

Urban growth, expansion of industrial, agricultural, recreational, and resource extractive activities, and renewable energy development raise concerns for lizard conservation in the Mojave Desert. We found evidence that two of the three species of common lizards were sensitive to land use. In particular, our maps of predicted density show greater variability in habitat quality in the more developed western side of the study area (Figures 4, 5, 6). Furthermore, although *C. draconoides* may be relatively well suited to higher temperatures, our results suggest it will be impacted by development that removes or degrades habitat. Retaining pockets of natural habitat and enhancing the quality of vegetation cover in developed areas may be important mechanisms for mitigating impacts to lizards and other wildlife (Ackley et al., 2015; Sullivan, Vardukyan, & Sullivan, 2014).

Desert ecosystems and the wildlife they support are vulnerable to the combined effects of climate change and habitat degradation (Sinervo et al., 2010; Thompson et al., 2008). Although reptiles have been difficult to survey and thus often neglected in multispecies monitoring efforts (Gibbons et al., 2000), advances in noninvasive survey and robust analytical methods are making it more practical to include reptiles and other overlooked taxa in these programs. Our results demonstrate this trajectory for lizards and how reasonably precise estimates of total population size and maps of density can inform conservation. These methods are likely transferable to other desert ecosystems throughout the world.

## CONFLICT OF INTEREST

None declared.

## AUTHOR CONTRIBUTIONS

BJF designed and oversaw the study, analyzed the data, and wrote the paper. DSN led field implementation of surveys. DSN and GDC assisted with the development of the survey method and participated in surveys. CWB designed and oversaw implementation of JTNP surveys used in model validation. CWB, DSN, and GDC contributed to the manuscript and assisted with research supporting its writing.

## Data Availability

Upon publication of this manuscript, the R code and data used in modeling will be archived in Dryad. https://doi.org/10.5061/dryad.j1m1t3t.
