# Supplementary material for: Hierarchical distance sampling to estimate population sizes of common lizards across a desert ecoregion
Source: Ecol Evol. 2019 Feb 20;9(6):3046–58. doi: 10.1002/ece3.4780 (PMC6434545; doi:10.1002/ece3.4780)
Supplement: Supplementary file 1 [file ECE3-9-3046-s001.docx]

**SUPPLEMENTAL MATERIALS**

Hierarchical Distance Sampling to Estimate Population Sizes of Common Lizards Across a Desert Ecoregion.

Table S1. Distribution of wildlife survey sites by vegetation type throughout the portion of the Mojave Desert within California, USA, April–July 2016.

| National Vegetation Classification Division ^a^ | Example species | Number of survey sites | Average NDVI ^b^ | Average elevation (m) |
| --- | --- | --- | --- | --- |
| Interior flooded forest  Cool semi-desert scrub and grassland  Pinyon - juniper woodland and scrub  Temperate & Boreal freshwater marsh,  wet meadow and shrubland  Warm desert freshwater marsh and bosque  Warm desert scrub and grassland  Western interior brackish marsh,  playa and shrubland  Not classified | *Populus fremontii*  *Yucca brevifolia, Y. schidigera*  *Juniperus osteosperma, Pinus monophylla*  *Schoenoplectus americanus, Phragmites australis*  *Olneya tesota, Prosopis glandulosa*  *Larrea tridentata, Ambrosia dumosa*  *Atriplex polycarpa* | 3  34  5  3  16  140  24  4 | 1,789  1,510  1,930  1,559  1,240  1,198  1,060  1207 | 881  1,138  1,490  403  504  748  463  731 |

^a^ See Sawyer et al. (2009).

^b^ Normalized difference vegetation index. Computed as average value within 1 km^2^ surrounding each site

Table S2. Lizard species observed during visual encounter surveys at 229 sites throughout the portion of the Mojave Desert within California, USA, April–July 2016.

| Lizard species | Total number of observations | Number sites where observed | Average number of observations per survey |
| --- | --- | --- | --- |
| *Aspidoscelis tigris*  *Uta stansburiana*  *Callisaurus draconoides*  *Sceloporus magister*  *Dipsosaurus dorsalis*  *Crotaphytus bicinctores*  *Sceloporus occidentalis*  *Gambelia wislizenii*  *Phrynosoma platyrhinos*  *Sauromalus ater*  *Xantusia vigilis*  *Urosaurus graciosus* | 195  185  111  18  11  10  8  6  6  3  1  1 | 97  83  55  10  9  7  5  6  4  3  1  1 | 0.28  0.27  0.16  0.03  0.02  0.01  0.01  0.01  0.01  <0.01  <0.01  <0.01 |

S3 lizard_model.R Computer code for running hierarchical distance sampling models

in the R programming language.

S4 lizard_data.RData Data used by R code for running models.
